# Successful osimertinib rechallenge in a patient with advanced non-small cell lung cancer following osimertinib-induced interstitial lung disease after treatment with nivolumab

**DOI:** 10.1007/s10637-017-0471-y

**Published:** 2017-05-02

**Authors:** Nobuaki Mamesaya, Hirotsugu Kenmotsu, Toshiaki Takahashi

**Affiliations:** Division of Thoracic Oncology, Shizuoka Cancer Center Hospital, 1007 Shimonagakubo, Nagaizumi-cho, Sunto-gun, Shizuoka, 411-8777 Japan

**Keywords:** Lung cancer, EGFR mutation, EGFR tyrosine kinase inhibitor, Anti-PD1 antibody, Interstitial lung disease, Rechallenge

Dear Editor:

We have already reported a case of a 38-year-old woman with advanced non-small cell lung cancer harboring a sensitive epidermal growth factor receptor (EGFR) L858R mutation with T790 M who was treated with osimertinib, a third generation EGFR-tyrosine kinase inhibitor (EGFR-TKI), which induced interstitial lung disease (ILD) after treatment with nivolumab [1]. In that report, we mentioned that an anti-programmed cell death-1 (anti-PD1) antibody therapies may be a risk factor for EGFR-TKI-induced ILD. Herein, we describe the clinical course of a patient who was successfully treated with osimertinib rechallenge after osimertinib-induced ILD following treatment with nivolumab.

**Fig. 1:**
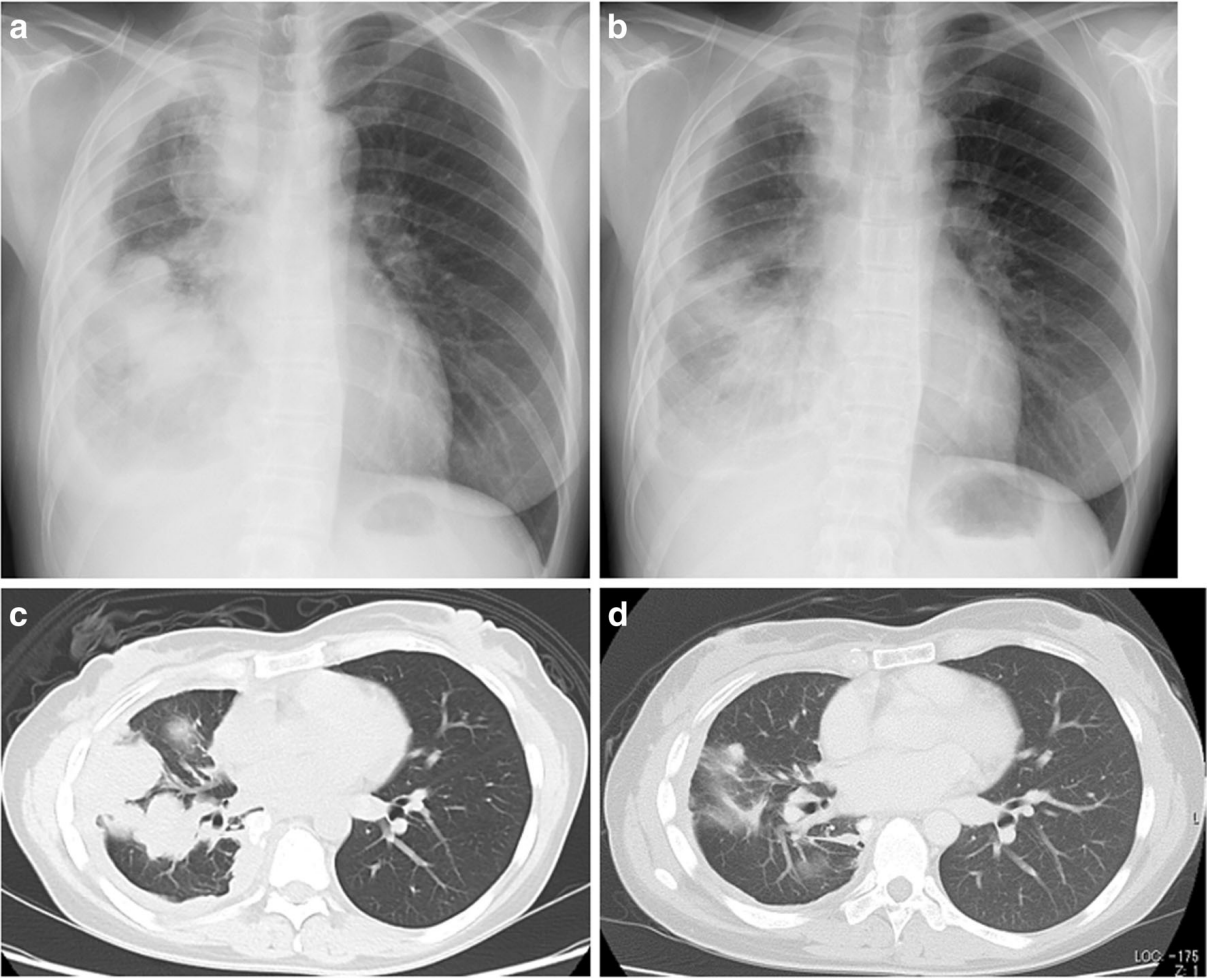
Chest radiographs before and after treatment. **a** Solid tumors with intrapulmonary metastasis have aggressive regrowth. **b** Remarkable shrinkage of tumors after osimertinib rechallenge treatment within 1 week. **c** Solid tumors with intrapulmonary metastasis and no evidence of preexisting interstitial pneumonia before osimertinib rechallenge treatment. **d** Remarkable shrinkage of tumors after osimertinib rechallenge treatment without evidence of the recurrence of osimertinib-induced interstitial lung disease

In our patient, osimertinib-induced ILD improved within 2 months after discontinuing osimertinib. She was treated with a combination of docetaxel and ramucirumab (10 mg/kg, up to four cycles), as sixth-line chemotherapy, from August 2016, and then she received gemcitabine monotherapy (up to two cycles). However, no significant response was observed in any conventional chemotherapy, and tumor-related pain became worsen regardless of the administration of oxycodone. Therefore, osimertinib rechallenge was considered based on the expected clinical benefit of its antitumor efficacy over the risk of osimertinib-induced ILD recurrence. Although the risk of drug-induced ILD recurrence may be increased by rechallenge, we considered that the interaction between EGFR-TKI and the anti-PD1 antibody was decreased, because the interval from treatment with nivolumab had been more than 6 month. After obtaining informed consent, a rechallenge of osimertinib (80 mg, once daily) was started as eighth-line treatment for 8 months after the last initiation of nivolumab. One week after the re-administration of osimertinib concurrently without a steroid, her symptom (right chest pain) improved, and right tumor shadows were shrinking, as indicated by the chest radiograph (Fig. 1a, b). Furthermore, 3 months after treatment with osimertinib, the chest computed tomography scan showed a remarkable antitumor response with improvement in right pleural thickening without evidence of the recurrence of osimertinib-induced ILD (e.g., bilateral diffuse, faint, ground-glass opacities or consolidation) (Fig. 1c, d). Now, she continues to receive treatment with osimertinib.

To our knowledge, this is the first case report of a successful osimertinib rechallenge following osimertinib-induced ILD after treatment with nivolumab. The strategy for treating lung cancer harboring a sensitive EGFR mutation is preferentially to administer EGFR-TKI. However, most patients unfortunately acquire resistance to EGFR-TKI. Several conventional chemotherapies or anti-PD1 therapies, in general, are initiated after resistance without a T790 M mutation to the first or second generation EGFR-TKI or the acquisition of osimertinib-resistance. In particular, PD1 antibody therapies are expecting to be one of the potential salvage chemotherapies. Kotake et al. reported a high frequency of osimertinib-induced ILD after immediate prior treatment with nivolumab in a series of 19 patients with EGFR-T790 M mutation-positive advanced non-small cell lung cancer who acquired resistance with the T790 M mutation [2]. They also showed that the administration interval between prior nivolumab and osimertinib tended to be shorter in patients with ILD than in those without ILD. However, it is unknown how long of an interval is safe for ILD due to the synergistic reaction between osimertinib and nivolumab. Brahmer et al. reported that the nivolumab serum half-life concentration (t1/2) was approximately 12 days (3 mg/kg dose), according to flow cytometric methods used to evaluate the pharmacodynamics of infused nivolumab [3]. In addition, they showed that estimating the plateau PD-1 occupancy on circulating CD3^+^ T cells after one infusion was observed at 4 to 24 h and ≥57 days. These data showed the high affinity of nivolumab for PD-1 in vitro, which suggests that sufficient concentrations persist to plateau PD-1 occupancy, even when nivolumab serum levels are undetectable (<1.2 μg/mL). However, it is unknown whether these findings in circulating lymphocytes reflect PD-1 occupancy on lymphocytes in the tumor or other normal tissues relating to antitumor activity or the development of immune-related adverse effects. This case report suggests that even if patients receive nivolumab therapy, a long interval from the last administration of nivolumab may reduce the risk of osimertinib-induced ILD.
